# Clinical Efficacy of Percutaneous Image-Guided Ablation in Breast Cancer Metastases to the Liver

**DOI:** 10.3390/cancers17233823

**Published:** 2025-11-28

**Authors:** Govindarajan Narayanan, Elizabeth Mary Ruiz, Madelon Dijkstra, Nicole T. Gentile, Danielle Donahue, Ripal T. Gandhi, Reshma L. Mahtani, Starr Mautner, Bente A. T. van den Bemd

**Affiliations:** 1 Herbert Wertheim College of Medicine, Florida International University, Miami, FL 33199, USA; govindarajann@baptisthealth.net (G.N.);; 2 Department of Interventional Oncology, Miami Cancer Institute, Baptist Health South Florida, Miami, FL 33176, USA; elizabeth.ruiz01@baptisthealth.net (E.M.R.); dd1989@mynsu.nova.edu (D.D.); 3 Department of Interventional Radiology, Miami Cardiac and Vascular Institute, Baptist Health South Florida, Miami, FL 33176, USA; 4 Department of Radiology and Nuclear Medicine, Amsterdam UMC, Location VUmc, Cancer Center Amsterdam, 1081 HV Amsterdam, The Netherlands; m.dijkstra3@amsterdamumc.nl; 5 Dr. Kiran C. Patel College of Allopathic Medicine, Nova Southeastern University, Fort Lauderdale, FL 33314, USA; 6 Department of Breast Medical Oncology, Miami Cancer Institute, Baptist Health South Florida, Miami, FL 33176, USA; 7 Department of Breast Surgical Oncology, Miami Cancer Institute, Baptist Health South Florida, Miami, FL 33176, USA

**Keywords:** breast cancer liver metastases, microwave ablation, irreversible electroporation, interventional oncology

## Abstract

**Objectives**: This single-center retrospective study evaluates the clinical efficacy and safety of percutaneous ablation using microwave ablation (MWA) and irreversible electroporation (IRE) in patients with breast cancer liver metastases (BCLM). **Methods**: Between August 2018 and December 2023, 32 patients underwent 40 image-guided ablations for 57 BCLM. Mean age was 61.3 years (range: 32–85), and mean tumor size was 2.9 cm (range: 0.9–7.0 cm). Fifty lesions were treated with MWA and seven with IRE. Clinical efficacy was assessed by m-RECIST response at first follow-up imaging (after ≥1 month) and by monitoring local tumor progression (LTP), local tumor progression-free survival (LTPFS), and overall survival (OS). Safety was evaluated by adverse events according to CTCAE. Kaplan–Meier statistics were used for LTPFS and OS. **Results**: Median follow-up was 32.4 months (95% CI 16.6–48.0). Complete response was observed in 34 tumors (59.6%), partial response in 14 (24.6%), and progressive disease in 9 (15.8%). LTP occurred in 37 tumors (64.9%), with a median time to progression of 11.1 months (95% CI 1.4–20.8). One- and two-year LTPFS rates were 43.6% and 34.1%. Sixteen patients died during follow-up, with median OS of 27.8 months (95% CI 19.0–36.6) and 1- and 2-year OS rates of 90.1% and 55.9%. No major complications occurred. Complications included eight Grade 1 and two Grade 2 complications. **Conclusions**: Percutaneous ablation demonstrates clinical efficacy and a favorable safety profile in selected BCLM patients, achieving OS comparable to the current literature. Further studies should clarify its additive role within multimodality treatment.

## Simple Summary

This study focuses on evaluating the clinical efficacy and safety of percutaneous ablation techniques, specifically microwave ablation (MWA) and irreversible electroporation (IRE), as treatment options for patients with liver metastases from breast cancer (BCLM) who are not eligible for surgery. While systemic therapies are standard for BCLM, patients with limited liver disease or progression under treatment may benefit from local therapies that can help control the disease, delay progression, and improve survival. The study finds that percutaneous ablation shows promising disease control and a favorable safety profile, with relatively low complication rates and good survival outcomes compared to other treatments. These results support further exploration of percutaneous ablation as a potential alternative in managing liver-dominant BCLM.

## 1. Introduction

Breast cancer is the most common malignancy among women worldwide and a leading cause of cancer-related mortality [1]. Despite advances in systemic therapies—including endocrine therapy, chemotherapy, targeted anti-HER2 agents, and immunotherapy—metastatic breast cancer (MBC) remains rarely curable [1]. The liver is a common metastatic site, affecting up to 15% of MBC patients, and is associated with poor prognosis [2,3]. Median overall survival (OS) after breast cancer liver metastases (BCLM) diagnosis ranges from 4 to 21 months, even with modern systemic treatment [4,5].

Systemic therapy remains the standard for most BCLM patients. However, a distinct subgroup with oligometastatic disease (≤5 lesions) or liver-dominant progression under systemic therapy may benefit from local treatments, which can sustain systemic control, delay progression, and potentially prolong survival. Surgical resection offers favorable long-term outcomes but is often unfeasible due to extrahepatic disease, comorbidities, or anatomical constraints [2,6]. For ineligible patients, minimally invasive local therapies—such as stereotactic body radiotherapy (SBRT), intra-arterial therapies (TACE, TARE/Y-90), or percutaneous ablation—are options [6,7,8]. 

Treatment choice depends on tumor size, number, location, and liver function. Intra-arterial therapies are generally palliative and repeatable, while percutaneous ablation offers curative potential with safe repeatability in case of recurrence. Microwave ablation (MWA) produces large, predictable zones less affected by the heat-sink effect, whereas irreversible electroporation (IRE), a non-thermal technique, enables treatment of tumors adjacent to critical structures such as bile ducts or vessels [9,10,11]. Both MWA and IRE have proven safe and effective in hepatocellular carcinoma and colorectal liver metastases (CRLM) [11]. The role of ablation in BCLM remains less clearly defined. Both the European Society for Medical Oncology (ESMO) and National Comprehensive Cancer Network (NCCN) guidelines primarily emphasize systemic therapies for metastatic breast cancer, with limited recommendations for locoregional treatments [8,12]. In contrast, CRLM management increasingly incorporates ablation into standard care based on prospective trials like COLLISION [13]. Data for BCLM are lacking, highlighting the need for further study.

This study evaluates the clinical efficacy and safety of percutaneous ablation (MWA and IRE) in BCLM patients with oligometastatic disease or progression under systemic therapy, where surgery is not feasible and systemic treatment alone is insufficient.

## 2. Materials and Methods

This retrospective study, conducted at a single institution, was carried out at Baptist Health South Miami in Florida. Data reporting adheres to the ‘Strengthening the Reporting of Observational Studies in Epidemiology’ (STROBE) guidelines [14]. The study has received approval from the institutional review board (2024-RETRO-NAR-002).

### 2.1. Data Collection

A retrospective chart review was conducted on BCLM patients who underwent percutaneous MWA or IRE, with at least one follow-up imaging exam post-ablation. The decision to offer ablation was made within a multidisciplinary tumor board, which included experts in medical oncology, interventional radiology, surgery, and radiation oncology. The tumor board evaluated each case based on several tumor factors, liver function, and overall performance status, as well as the patient’s response to prior systemic therapies. In particular, ablation was considered for patients with oligometastatic disease (≤5 lesions) or progression under systemic therapy when surgery was not feasible due to factors such as extrahepatic disease, anatomical constraints, or comorbidities. Other local therapies such as SBRT were typically chosen for patients who were not candidates for surgery or ablation but required focused tumor control. Intra-arterial therapies (TACE, TARE/Y-90), were reserved for patients with more advanced diseases or those with extensive or unresectable liver metastases, where a palliative approach was necessary. The choice between MWA or IRE ablation was then made based on size and proximity to critical structures and the expertise of the operator. The decision to continue, adjust, or change systemic and/or hormonal therapy, alongside and after ablation, was based on prior treatments, resistance patterns, and the patient’s genetic and receptor status.

### 2.2. Procedures

#### 2.2.1. MWA

All percutaneous MWA procedures were performed using commercially available microwave systems (Emprint Ablation System, Medtronic, Minneapolis, MN, USA; Neuwave Ablation System, Ethicon, Johnson & Johnson, Cincinnati, OH, USA; Solero Microwave Ablation System, AngioDynamics, Latham, NY, USA), which include a generator, an internally water-cooled antenna, and 14- to 17-gauge probes. Ablation was carried out under general anesthesia using CT guidance, after percutaneous probe placement into the tumor. Microwave energy was typically delivered at 40–100 W for 5–10 min, depending on tumor size. If peri-procedural CT suggested incomplete ablation, the probe was repositioned to achieve adequate margins. In accordance with CIRSE standards for thermal ablation of liver tumors, technical success was defined as achieving a minimum tumor-free ablation margin of 5 mm, with a target margin of greater than 1 cm whenever feasible [15,16].

#### 2.2.2. IRE

All percutaneous IRE procedures were performed under general anesthesia using the NanoKnife™ system (AngioDynamics, Queensbury, NY, USA), which includes a generator, monopolar probes, and the AccuSync device. Depending on tumor size, 2 to 4 monopolar probes were placed with a typical spacing of 1.5–2.3 cm to ensure adequate electric field coverage and minimize the risk of incomplete ablation. Probe positioning was guided by CT imaging combined with 3D reconstruction. The AccuSync device synchronized pulse delivery with the patient’s cardiac R-waves to reduce the risk of ventricular arrhythmia. IRE was contraindicated in patients with a history of cardiac arrhythmias or if a safe trajectory for probe placement could not be achieved.

### 2.3. Imaging and Follow-Up

Pre-ablation contrast-enhanced CT was performed for treatment planning, assessing tumor characteristics and critical structures. Procedures were guided by CT-fluoroscopy, and post-ablation contrast CT confirmed complete ablation and the absence of complications. Patients were monitored in the recovery area for at least 4 h and examined the following day with routine laboratory tests. Discharge was allowed once clinically stable. Follow-up imaging was performed at approximately 1, 3, 6, and 12 months post-procedure, and thereafter as clinically indicated.

### 2.4. Objectives

The primary endpoint of this study was clinical efficacy, assessed by treatment response at the first follow-up imaging, according to the modified Response Evaluation Criteria in Solid Tumors (mRECIST) criteria [17]. Tumor response was evaluated by comparing the reduction in enhancement on diffusion-weighted MRI or changes in 18F-FDG uptake on PET-CT, conducted at least 1 month after ablation. A complete response was defined as the absence of enhancement or 18F-FDG avidity, while a partial response indicated incomplete reduction. Stable disease was characterized by no change, and progressive disease was identified by an increase in enhancement or focal 18F-FDG avidity at the tumor site. Local tumor progression (LTP) was defined as an unequivocally enlarging mass or focal 18F-FDG avidity at or near the outer boundaries of the treated tumor during follow-up. Local tumor progression-free survival (LTPFS) and overall survival (OS) were assessed using the Kaplan–Meier survival curves over the total follow-up period. Censored cases, including patients who were lost to follow-up, withdrew, or were still alive at the end of the study without progression, were included in the analysis. Censoring was accounted for by noting the time of the last available follow-up. Radiologic evaluation was performed by an experienced radiologist, with independent review for accuracy when necessary. Secondary endpoints included procedure-related safety, evaluated by adverse events according to the Common Terminology Criteria for Adverse Events (CTCAE) [18] and length of hospital stay. Data analysis was performed using Excel (Microsoft), SPSS^®^ Version 28.0 (IBM^®^, Armonk, New York, NY, USA), and R version 4.2.1 (R Foundation, Vienna, Austria) [19,20].

## 3. Results

### 3.1. Patient and Tumor Characteristics

Between August 2018 and December 2023, 32 patients underwent 40 treatments of image-guided percutaneous ablation under general anesthesia for 57 BCLM. Patient oncologic information and procedure and tumor characteristics are summarized in Table 1 and Table 2, respectively. Mean age was 61.3 years at time of treatment (range: 32–85), and mean size of treated lesions was 2.9 cm (range: 0.9–7.0 cm). Synchronous metastases were found in 25%, and stable extrahepatic disease was present in 84.0% of the cases. The majority of the patients (43.8%) were undergoing third-line therapy when ablation was considered as a local treatment. Hormone receptors were categorized as estrogen receptors (ER; positive in 93.8% of cases), progesterone receptors (PR; positive in 78.1% of cases), and human epidermal growth factor receptor 2 (HER2; positive in 25.0% of cases). Twenty patients (62.5%) had subtype luminal A (ER+/PR+/HER2−). Fifty lesions were treated with MWA and seven with IRE with a median follow-up of 32.4 months (95% CI 16.6–48.0). Regarding systemic treatment, adjuvant chemotherapy for primary breast cancer was used in 65.5% of the cases, adjuvant hormone therapy in 59.4%, adjuvant targeted therapy in 6.3%, and adjuvant radiotherapy in 46.9%. Neoadjuvant chemotherapy for metastases was used in 68.8% of the cases, neoadjuvant hormone therapy in 90.6%, and neoadjuvant targeted therapy in 90.6%. Locoregional therapy for distant metastases was performed in 4 patients.

### 3.2. Efficacy

**Figure 1:**
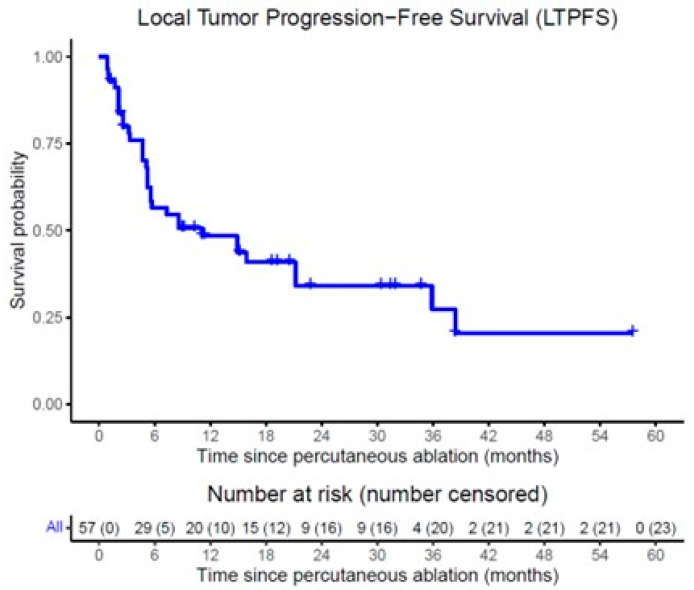
Kaplan–Meier survival curves of local tumor progression-free survival (LTPFS) following percutaneous ablation (MWA or IRE) per tumor.

**Figure 2:**
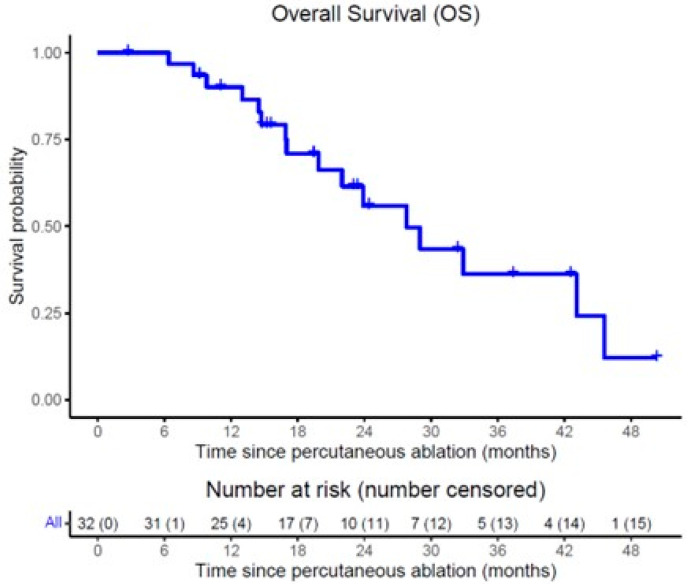
Kaplan–Meier survival curves of overall survival (OS) following percutaneous ablation (MWA or IRE) per patient.

**Figure 3:**
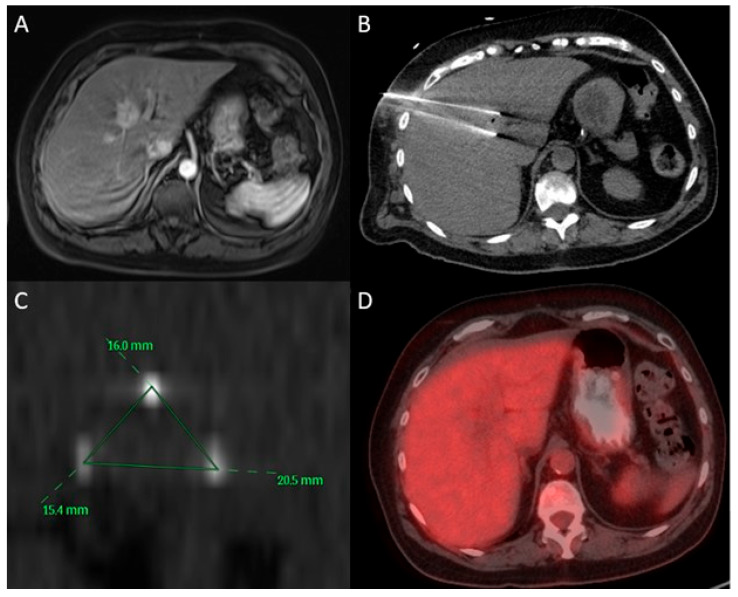
A 67-year-old female with a history of bilateral breast cancer (right invasive lobular carcinoma, ER/PR positive, HER2 negative), diagnosed in 2015, status post mastectomy and adjuvant chemotherapy. She developed bone metastases in 2021 and is being treated with Xeloda and Fulvestrant. (**A**) Abdominal MRI demonstrates an enhancing, diffusion-restricting mass within segment 8/5, partially extending into 4a/4b, measuring approximately 3.4 × 2.4 cm. (**B**,**C**) Intraprocedural CT showing intermittent placement of three 17-gauge NanoKnife IRE ablation needles. (**D**) Follow-up PET-CT at 3 months reveals a calcified granuloma in the right hepatic lobe with no evidence of focal FDG-avid lesions in the liver.

**Figure 4:**
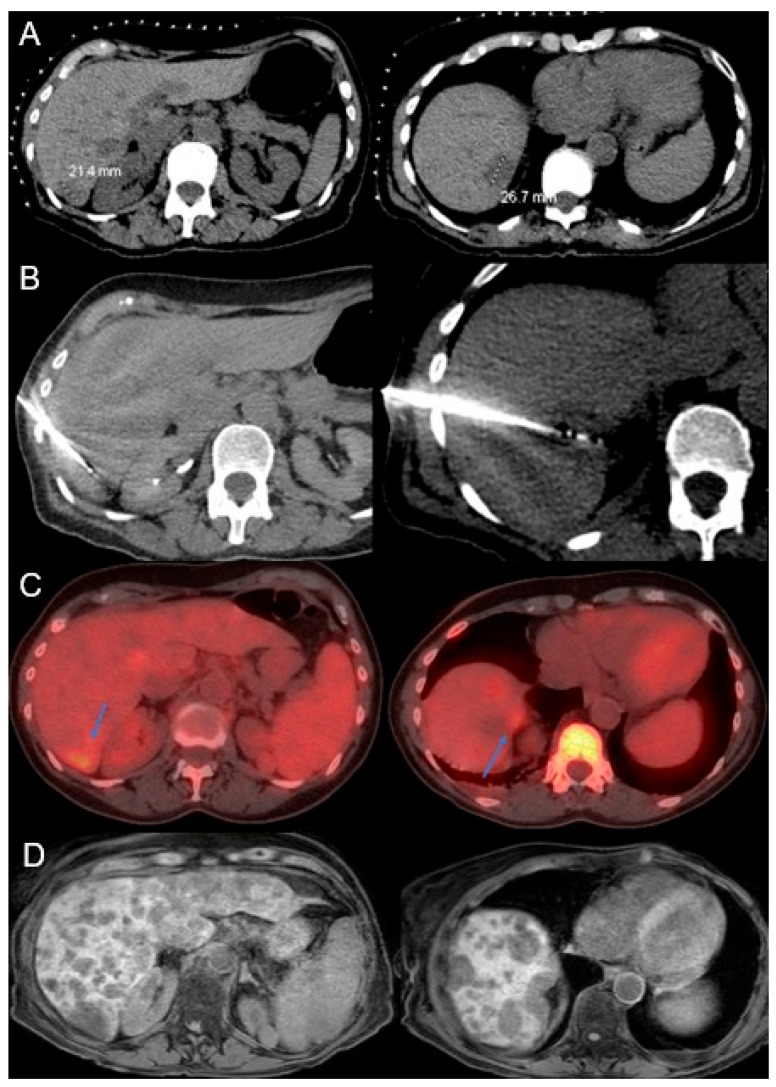
A 68-year-old female with recurrent hormone-negative, HER2-positive left breast cancer and liver-dominant metastases. Initially treated with chemotherapy including Herceptin, showing partial response. (**A**) Pre-procedural CT shows lesions in segment 6 (2.1 cm) and segment 7 (2.7 cm). (**B**) Patient underwent MWA with intra-procedural CT showing the MWA probes within the lesions. A complete response was seen on first follow-up MRI. (**C**) PET-CT at 5 months reveals increased FDG uptake in the ablation zones (blue arrows), suggesting recurrence. Subsequently treated with Y-90 radioembolization via the right hepatic artery. (**D**) One-month follow-up shows widespread metastatic disease and impaired liver function. Patient is on systemic therapy and may become eligible for repeat Y-90 if response improves. This case illustrates the aggressive nature of BCLM.

On follow-up imaging, complete response to treatment was observed in 34 tumors (59.6%), partial response in 14 tumors (24.6%), and progressive disease in 9 tumors (15.8%). LTP occurred in 37 tumors (64.9%), with a median time to LTP of 11.1 months (95% CI: 1.4–20.8) and 1- and 2-year LTPFS of 43.6% and 34.1%, respectively, by Kaplan–Meier statistics shown in Figure 1. During follow-up, 16 of the 32 patients died. Median OS from ablation was 27.8 months (95% CI: 19.0–36.6), with 1- and 2-year OS of 90.1% and 55.9%, respectively, by Kaplan–Meier statistics depicted in Figure 2. An example case is shown in Figure 3, with Figure 4 further illustrating the aggressive nature of BCLM.

### 3.3. Safety

Of the 40 image-guided ablation procedures performed, 30 were completed without any immediate complications. Eight treatments (20%) resulted in Grade 1 complications, including six cases of small, self-limiting hematomas identified on follow-up imaging and two cases of right upper abdominal or shoulder pain that required an additional day of hospitalization. Two procedures (5%) led to Grade 2 complications: one case of right lobe pneumonia with pain necessitating five days of intravenous antibiotics and analgesia, and one case of a small lower-lobe pneumothorax that did not require chest tube placement. No major complications occurred. Thirty-four patients (85%) were discharged after an overnight stay, while six remained hospitalized for two nights.

## 4. Discussion

BCLM remains a challenging clinical problem with limited curative options; surgical resection offers the best long-term outcomes but is frequently not feasible because of extrahepatic disease, comorbidity, or unfavorable anatomy [2,6]. In our selected cohort—characterized by a substantial proportion (62.5%) of patients with ablation indicated due to progression under systemic therapy—percutaneous ablation, predominantly MWA with a minority treated using IRE, achieved disease control in 84.2% of treated lesions at first follow-up, with a median LTPFS of 11.1 months (95% CI: 1.4–20.8). This result aligns with the limited evidence on percutaneous ablation for BCLM, primarily based on small RFA series reporting LTP rates of 10–40% in highly selected patients with smaller lesions [21,22,23,24,25]. Importantly, the frequent progression under systemic therapy observed in our cohort reflects the aggressive biology of tumors in this vulnerable population, limiting treatment options and contributing to surgery ineligibility.

The median OS in our cohort was 27.8 months (95% CI 19.0–36.6), with 1- and 2-year OS rates of 90.1% and 55.9%, respectively. While promising, these outcomes may be influenced by the predominance of ER+/HER2− (luminal A) tumors, which make up 62.5% of the cohort and are associated with a more indolent disease course and better prognosis. This likely contributed to the favorable OS, despite the fact that most patients had progression under systemic therapy prior to ablation. These outcomes compare favorably with other local therapies for BCLM, underscoring the potential clinical benefit of liver-directed percutaneous ablation in appropriately selected patients [26]. For reference, surgical resection has reported median OS of 26–50 months and 5-year OS rates of 20–40% [2,27,28,29], and SBRT yields 1- and 2-year OS rates of 85% and 57%, respectively [30]. In contrast, TACE and TARE (Y-90), which are generally reserved for patients with more advanced disease, yield median OS of 15–20 months and 10–15 months, respectively [31,32,33,34]. These data suggest that percutaneous ablation, when used for liver-dominant or oligometastatic BCLM patients ineligible for surgery, can yield OS outcomes approaching those of surgery and SBRT, while intra-arterial therapies remain valuable palliative options for more advanced disease. Importantly, the favorable survival in our cohort was observed despite the presence of stable extrahepatic disease in most patients (84%), supporting the concept that local control of dominant hepatic lesions can contribute to prolonged systemic stability and survival. However, these results must be interpreted in light of inherent selection bias in retrospective cohorts and the potential influence of tumor biology, such as the predominance of luminal A tumors. A key limitation of our study is the lack of direct comparisons between ablation and other local therapies, such as SBRT. Few studies have compared these treatments for BCLM, and the ongoing COLLISION-XL trial, which compares SBRT and MWA for unresectable CRLM (3–5 cm) [35], highlights the need for similar research in BCLM to optimize treatment strategies.

In our series of 40 image-guided ablations, complication rates were low, with only two Grade 2 events (5%) and no major complications. Most adverse events were mild and self-limiting, aligning with reported safety profiles of thermal ablation. While surgical resection remains the gold standard for local control in BCLM, it is associated with higher morbidity and longer hospital stays, as confirmed by the COLLISION trial for CRLM, which showed that thermal ablation offered superior local control, fewer complications, and shorter hospital stays while being non-inferior to surgery in terms of overall survival [13,27,36]. The short hospital stays (mostly one night) and absence of major complications highlight the favorable safety and recovery profile of percutaneous ablation, making it an ideal option for patients unfit for surgery or with limited disease.

This study has important limitations. Its retrospective, single-center design limits causal inference and generalizability. The median follow-up was 32 months, suitable for medium-term outcomes, but limited follow-up restricts long-term survival or late-onset event assessment. The cohort’s heterogeneous characteristics and varied treatment modalities—mostly MWA with a smaller IRE subgroup—complicate direct comparisons and outcome interpretation. The absence of a control group and relatively small sample size, especially in the IRE subgroup, further limit statistical power. Selection bias is inherent, as patients were chosen based on ineligibility for surgery and other clinical factors, which may influence survival and treatment responses. The predominance of luminal A tumors likely contributed to the high survival rates. Future studies should account for subtype distribution, as this may affect the clinical benefit of liver-directed therapies like ablation. Additionally, inclusion of tumors near critical structures, often treated with IRE, and larger tumor sizes may have contributed to higher observed local tumor progression rates. Future prospective multicenter studies with larger, more homogeneous populations and control groups are needed to validate these findings and clarify the role of percutaneous ablation in managing BCLM.

## 5. Conclusions

In selected patients with BCLM who are not surgical candidates, percutaneous ablation demonstrates promising clinical efficacy and a favorable safety profile. Our findings support further prospective evaluation of liver-directed ablation (MWA and IRE) within standardized, multicenter protocols and comparative studies against other liver-directed therapies.

## Figures and Tables

**Table 1:** Patient characteristics.

Patient Characteristics (N = 32)		Value (N, %)
Age in years (mean, range)		61.3 (32–85)
Extrahepatic disease	Total	27 (84%)
	Bone	24 (75%)
	Brain	6 (18.8%)
	Lung	7 (21.9%)
	Ovaria	1 (3.1%)
	Distant lymph nodes	6 (18.8%)
Synchronous metastases		8 (25%)
Indication for ablation	Oligometastatic disease	12 (37.5%)
	Progression under systemic therapy	20 (62.5%)
Line of therapy at time of ablation	1	2 (6.3%)
	2	7 (21.95)
	3	14 (43.8%)
	4	5 (15.6%)
	5	2 (6.3%)
	6	2 (6.3%)
Receptor status	ER+	30 (93.8%)
	PR+	25 (78.1%)
	HER2+	8 (25.0%)
Adjuvant therapy for breast primary	Surgery breast primary	31 (96.9%)
	Radiotherapy	15 (46.9%)
	Chemotherapy	21 (65.6%)
	Hormonal therapy	19 (59.4%)
	Targeted therapy	2 (6.3%)
Neoadjuvant therapy for metastases before ablation	Locoregional therapy for other metastases	4 (12.5%)
	Chemotherapy	22 (68.8%)
	Hormonal therapy	29 (90.6%)
	Targeted therapy	29 (90.6%)

ER = estrogen receptor; PR = progesterone receptor; HER2 = human epidermal growth factor receptor 2.

**Table 2:** Procedure and tumor characteristics.

Procedure and Tumor Characteristics (N = 57)		Value (N, %)
Type of procedure	MWA	50 (87.7%)
	IRE	7 (12.3%)
Number of liver tumors per procedure	1	26 (65.0%)
	2	11 (27.5%)
	3	3 (7.5%)
Location of tumor (segment)	1	1 (1.8%)
	2	5 (8.8%)
	3	7 (12.3%)
	4 a/b	12 (21.1%)
	5	7 (12.3%)
	6	6 (10.5%)
	7	11 (19.3%)
	8	8 (14.0%)
Largest tumor diameter size in cm (mean, range)		2.9 (0.9–7.0)

MWA = microwave ablation; IRE = irreversible electroporation.

## Data Availability

Data is available upon request from Baptist Health South Miami.

## Abbreviations

The following abbreviations are used in this manuscript:

BCLM	Breast Cancer Liver Metastases
MBC	Metastatic Breast Cancer
OS	Overall Survival
SBRT	Stereotactic Body Radiotherapy
TACE	Transarterial Chemoembolization
TARE/Y-90	Transarterial Radioembolization/Yttrium-90
MWA	Microwave Ablation
IRE	Irreversible Electroporation
CT	Computed Tomography
CIRSE	Cardiovascular and Interventional Radiological Society of Europe
m-RECIST	Modified Response Evaluation Criteria in Solid Tumors
LTP	Local Tumor Progression
LTPFS	Local Tumor Progression-Free Survival
CTCAE	Common Terminology Criteria for Adverse Events
RFA	Radiofrequency Ablation
CRLM	Colorectal Liver Metastases
ESMO	European Society for Medical Oncology
NCCN	National Comprehensive Cancer Network
